# Biological Diagnosis of Alzheimer’s Disease Based on Amyloid Status: An Illustration of Confirmation Bias in Medical Research?

**DOI:** 10.3390/ijms242417544

**Published:** 2023-12-16

**Authors:** Benoît Souchet, Alkéos Michaïl, Baptiste Billoir, Jérôme Braudeau

**Affiliations:** AgenT SAS, 4 Rue Pierre Fontaine, 91000 Evry-Courcouronnes, France; benoit.souchet@agent-biotech.com (B.S.); alkeos.michail@agent-biotech.com (A.M.); baptiste.billoir@agent-biotech.com (B.B.)

**Keywords:** Alzheimer’s disease, amyloid diagnostic value, confirmation bias, biomarkers

## Abstract

Alzheimer’s disease (AD) was first characterized by Dr. Alois Alzheimer in 1906 by studying a demented patient and discovering cerebral amyloid plaques and neurofibrillary tangles. Subsequent research highlighted the roles of Aβ peptides and tau proteins, which are the primary constituents of these lesions, which led to the amyloid cascade hypothesis. Technological advances, such as PET scans using Florbetapir, have made it possible to visualize amyloid plaques in living patients, thus improving AD’s risk assessment. The National Institute on Aging and the Alzheimer’s Association introduced biological diagnostic criteria in 2011, which underlined the amyloid deposits diagnostic value. However, potential confirmation bias may have led researchers to over-rely on amyloid markers independent of AD’s symptoms, despite evidence of their limited specificity. This review provides a critical examination of the current research paradigm in AD, including, in particular, the predominant focus on amyloid and tau species in diagnostics. We discuss the potential multifaceted consequences of this approach and propose strategies to mitigate its overemphasis in the development of new biomarkers. Furthermore, our study presents comprehensive guidelines aimed at enhancing the creation of biomarkers for accurately predicting AD dementia onset. These innovations are crucial for refining patient selection processes in clinical trial enrollment and for the optimization of therapeutic strategies. Overcoming confirmation bias is essential to advance the diagnosis and treatment of AD and to move towards precision medicine by incorporating a more nuanced understanding of amyloid biomarkers.

## 1. A Concise Historical Account of Alzheimer’s Disease Discoveries (Figure 1)

Alzheimer’s disease (AD), first identified in 1906, marked a turning point in neuroscientific research. Dr. Alois Alzheimer, a German psychiatrist and neuropathologist, was seminal in elucidating this pathology, which is characterized by progressive memory loss and cognitive decline. He described Auguste Deter’s case, a patient exhibiting severe memory impairment. Post-mortem analysis of Deter’s brain revealed critical neuropathological lesions: amyloid plaques and neurofibrillary tangles [1]. These findings, initially reported in 1906, were fundamental for establishing the neuropathology of AD. Yet, comprehensively understanding this complex condition required decades of dedicated research, which gradually unveiled the multifaceted nature of its pathogenesis.

**Figure 1:**
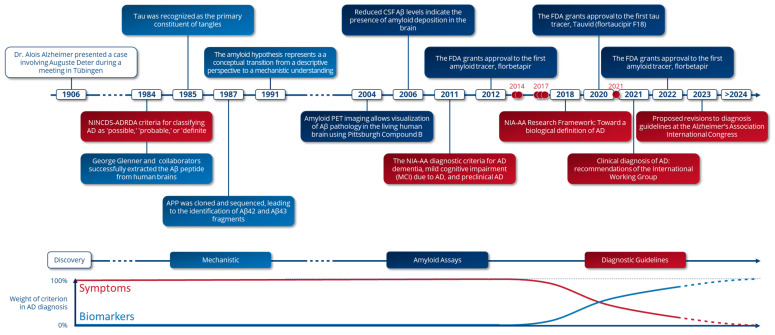
Alzheimer’s disease diagnosis research timeline. Major advances that have shaped AD diagnosis. Red circles show Cochrane review publications determining the diagnostic test accuracy of cerebrospinal fluid (CSF) assays or amyloid PET imaging in detecting AD in patients with mild cognitive impairment (MCI) or dementia. The light blue boxes correspond to advances in basic and mechanistic research, the dark blue boxes to technical advances in biomarker assays, and the dark red boxes to proposed recommendations for Alzheimer’s diagnosis.

In 1984, George Glenner and Colin Masters isolated the Aβ peptide, a key component of amyloid plaques in AD [2]. This discovery marked a significant advancement in understanding the disease’s pathology. The following year, researchers identified the tau protein as a main component of neurofibrillary tangles in AD patients, thus further elucidating the disease’s molecular basis [3,4,5,6]. In 1987, the cloning and sequencing of the APP gene revealed important peptide fragments, like Aβ42 and Aβ43, which were found to be neurotoxic [7,8,9,10]. A significant breakthrough came in 1991 with John Hardy’s amyloid cascade hypothesis. This hypothesis linked soluble Aβ peptide to tau hyper-phosphorylation, amyloid plaques, tangle formation, and subsequent neuronal dysfunction, thereby offering a mechanistic view of AD progression [11].

Research over time has revealed that amyloid plaques and neurofibrillary tangles precede the clinical symptoms of AD dementia. This understanding spurred the development of technologies for early detection of these brain lesions. A significant milestone was achieved in 2004 with the advent of positron emission tomography (PET) imaging using Pittsburgh Compound B (PiB), thus enabling the in vivo visualization of Aβ deposits in patient brains, a critical advancement in AD diagnostics [12]. This development was not only pivotal in allowing direct observation of disease progression but also for enhancing our understanding of its pathophysiology. Complementary to this, cerebrospinal fluid (CSF) biomarkers emerged as significant diagnostic tools. In 2006, a notable correlation was identified between decreased soluble Aβ42 levels in CSF and increased accumulation of amyloid proteins in the brain [13], thus providing a valuable approach for tracking disease progression.

In 2012, the FDA approved the first amyloid tracer, Florbetapir (Amyvid, Eli Lilly, Indianapolis, IN, USA), [14] which facilitated the visualization of Aβ deposits in the human brain. Subsequently, the FDA approved the first tau tracer, Flortaucipir (Tauvid, Eli Lilly, Indianapolis, IN, USA) in 2020 [15], followed by novel amyloid quantification methods in cerebrospinal fluid (CSF), the Elecsys Amyloid Plasma Panel (Roche Diagnostics International Ltd., Rotkreuz, Switzerland), and the Elecsys^®^ Phospho-tau (181P) (Roche Diagnostics International Ltd, Rotkreuz, Switzerland) [16]. Further augmenting diagnostic capabilities, additional assays for amyloid and tau biomarkers in CSF were approved, including the Lumipulse G β-Amyloid Ratio (Fujirebio, Inc., Tokyo, Japan, approved in 2022) [17] and the Elecsys^®^ Total-tau test (Roche Diagnostics International Ltd, Rotkreuz, Switzerland, approved in 2023) [16]. Moreover, Lumipulse^®^ G p-tau 181 (Fujirebio, Inc., Tokyo, Japan,) [18] and the Neurology 3-Plex A kit (Aβ40, Aβ42, tau) (Quanterix, Lexington, MA, USA) [19] are undergoing FDA approval or are available under CLIA regulations. These assays, which are currently being evaluated for their efficacy in measuring amyloid, tau, or p-tau concentrations in plasma, represent significant advancements in AD diagnostics and therapeutics. For example, plasma p-tau 217 assays are currently being evaluated for their ability to predict amyloid status as measured by PET scan or CSF analysis [20].

## 2. Advancing towards a Biological Framework for Defining Alzheimer’s Disease

In 1984, the NINCDS-ADRDA Work Group standardized the diagnostic criteria for AD dementia, introducing the concepts of “possible”, “probable”, and “definite” AD [20]. A confident clinical diagnosis of probable AD dementia can be established when there is a characteristic gradual onset of dementia with progression and there are no concurrent systemic or brain disorders that could explain the gradual decline in memory and other cognitive functions. The major component of the AD diagnosis is thus the symptoms and cognitive decline characteristic of AD, and the laboratory results (normal lumbar puncture as evaluated by standard techniques, normal pattern or nonspecific changes in electroencephalogram, such as increased slow-wave activity, and evidence of cerebral atrophy on computed tomography with progression documented by serial observation) are only intended to increase the clinician’s confidence in the AD diagnosis. 

The peptide Aβ42 in its soluble form, either as oligomers or protofibrils, is recognized as the primary toxic agent causing AD’s cognitive symptoms [21]. This makes it a very relevant biomarker for early detection before AD dementia onset in patients. However, the measurement of Aβ42 levels in the brain during a patient’s life is currently not feasible. The detection of these soluble forms has been limited to post-mortem biochemical examination of brains from individuals who exhibited AD dementia symptoms [2]. Consequently, AD diagnosis guidelines have recommended the assessment of amyloid accumulation in the brain as AD’s core biomarker. This is typically performed through amyloid PET imaging or by measuring the levels of Aβ42 and/or p-tau in the cerebrospinal fluid (CSF) [22,23]. In 2011, the National Institute on Aging and the Alzheimer’s Association (NIA-AA) orchestrated collaborative efforts that culminated in the publication of new diagnostic guidelines. These NIA-AA 2011 criteria for AD rest upon an evaluation of cognitive impairments complemented by the quantification of amyloid deposits within the brain. This quantification is achieved through measurements of Aβ42 peptides and/or p-tau protein in CSF or PET imaging [24] (NIA-AA diagnosis criteria, 2011). These criteria unveiled a diagnostic framework rooted in biology and encompassing dementia due to AD [22], Mild Cognitive Impairment (MCI) due to AD [23], and preclinical AD [25]. However, the methodologies tied to amyloid deposits entail invasive and non-scalable medical procedures. 

During the establishment of these diagnostic biological criteria, the authors illuminated certain concerns. They accentuated the imperative of extensive validation work for criteria employing biomarkers and the standardization of biomarker analysis, particularly in community settings [23]. Subsequent years witnessed a wealth of research into these fundamental biomarkers and their diagnostic significance. 

Amyloid PET imaging has shown significant effectiveness in differentiating AD patients from cognitively normal cases, and it has achieved a sensitivity of 91% and a specificity of 81% [26]. This level of performance is echoed in meta-analyses that examined either amyloid or tau biomarkers in the CSF, used alone or in combination, to distinguish AD patients with dementia from those without cognitive impairments. Specifically, the combined use of CSF Aβ42 and tau biomarkers was effective in identifying AD dementia, demonstrating 89% sensitivity and 87% specificity when compared to non-demented controls [27]. 

To further understand the differential diagnostic value of amyloid biomarker measurements using PET or CSF methods, both prospective and retrospective studies have been conducted. These studies involved participants at the MCI or dementia stages, with those at the MCI stage being clinically followed until their condition progressed to AD dementia or another form of brain disorder. The outcomes of these studies have been synthesized in Cochrane meta-analyses. In these meta-analyses, researchers calculated the diagnostic test accuracy of CSF assays or amyloid PET imaging when detecting individuals with mild cognitive impairment (MCI) [28,29,30,31,32,33] or dementia that will develop AD dementia symptoms [34]. Crucially, the diagnostic test accuracy was evaluated based on participants for whom the definitive diagnosis relied on clinical follow-up during prospective or retrospective studies, as opposed to relying solely on amyloid biomarker measurements [35,36].

**Figure 2:**
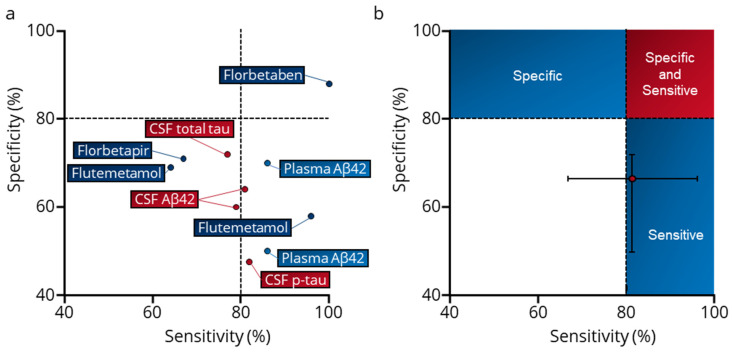
Overview of diagnostic test accuracy (DTA) for cerebrospinal fluid (CSF) assays, plasma assays or amyloid PET imaging in detecting Alzheimer’s disease among patients with mild cognitive impairment (MCI) or dementia, as determined through Cochrane reviews’ meta-analysis. (**a**) Specificity/sensitivity pair derived from Cochrane reviews for amyloid PET (dark blue circles); Aβ42, total tau, and p-tau assays in CSF (red circles); and plasma Aβ42 (light blue circles). (**b**) Median sensitivity (81.5%) and median specificity (66.5%) calculated based on performances reported in Cochrane reviews. Median ± 95% Confidence interval. Meta-analyses were searched using the Cochrane journal database searching engine with the keyword “Alzheimer”. We identified 110 Cochrane reviews with “Alzheimer” in the Title, Abstract, or Keywords. We then selected the reviews in the “diagnostic test accuracy (DTA)” topic, resulting in 18 reviews. Finally, only meta-analyses relating to amyloid or tau biomarkers were retained for analysis.

Cumulatively, these Cochrane reviews unveiled a diagnostic test accuracy range for biomarkers related to the deregulation of amyloid metabolism, which spanned between 64% and 100% for sensitivity and between 47.5% and 88% for specificity (Table 1) to predict the patients who will develop symptoms of AD dementia. The median performance of these tests, predicated on an estimate of amyloid plaque presence, stands at 81.5% (95% CI: 67–96%) for sensitivity and 66.5% (95% CI: 50–72%) for specificity (Figure 2). Consequently, given an AD prevalence of 60% among the population with cognitive impairment (MCI and dementia), the resulting false-positive rate from a diagnosis exclusively anchored in amyloid metabolism deregulation computes to 21.5%. This analysis reveals that 18.5% of patients progressing to AD dementia are amyloid negative, while 33.5% of individuals with non-AD brain disorders test positive for amyloid [37,38]. Incorporating amyloid biomarkers into cognitive assessments significantly improves the detection accuracy of prodromal AD patients who will develop AD dementia symptoms in the MCI population. This combined approach reduces the rate of false positives in the MCI cohort by 50% compared to the use of neuropsychological assessments alone. The integration of these biomarkers into clinical trial criteria has enabled clinicians to create participant groups enriched with prodromal AD patients compared to the use of cognitive criteria alone, thus improving the evaluation of anti-amyloid therapies on more targeted populations. Such strategic patient selection has been crucial in achieving significant advancements in clinical trials, particularly for pioneering treatments like lecanemab and donanemab.

In the absence of more specific biomarkers for predicting cognitive decline due to AD, the inclusion of patients with MCI due to AD in clinical trials has been a pragmatic and pivotal step forward. This strategy has contributed to the success of Phase III trials for at least two drugs. These treatments, which involve anti-amyloid antibodies, facilitate the clearance of soluble Aβ42 peptides and amyloid plaques. Moreover, CSF assays for amyloid and tau proteins, as well as PET imaging, have been instrumental as companion diagnostics. These tools have not only enabled the stratification of specific MCI patient subgroups but also allowed for in vivo monitoring of the engagement of anti-amyloid antibodies with their targets.

The Cochrane review meta-analyses focused on amyloid radiotracers, such as PIB, Florbetapir, and Flutamamol, which remain key tools used by clinicians for determining amyloid status via PET imaging. While these radiotracers have seen advancements, particularly in quantitative analysis, many neurological centers still employ visual quantification. Studies suggest no significant difference in diagnostic performance between visual and quantitative methods [26], thus affirming the ongoing relevance of Cochrane reviews for amyloid PET imaging.

For CSF biomarkers, recent advancements have been validated with amyloid status from PET imaging or autopsy as the benchmark. The Lumipulse CSF p-tau/Aβ1−42 assay (Fujirebio, Inc., Tokyo, Japan) showed an AUROC of 88% compared with Florbetapir [39]. The INNOTEST CSF Aβ42 (Fujirebio, Inc., Tokyo, Japan) achieved a sensitivity of 80% and a specificity of 82% for predicting neuritic plaque presence at autopsy [16]. The Elecsys CSF p-tau181/Aβ42 ratio (Roche Diagnostics International Ltd, Rotkreuz, Switzerland) reached an AUC of 0.94 versus the amyloid PET [16]. While CSF assays have been potentially improved since the Cochrane reviews’ publication, they cannot outperform the diagnostic performance of amyloid PET radiotracers because of their nearly perfect correlation with amyloid PET results, thus affirming the ongoing relevance of Cochrane reviews for amyloid and p-tau assays. This is supported by a recent publication evaluating the diagnostic precision of CSF biomarkers (specifically, Aβ42, total-tau, p-tau, and their ratios) measured using the fully automated CLEIA assay (Lumipulse, Fujirebio, Inc., Tokyo, Japan). The study reported AUCs ranging from 0.62 to 0.72 in differentiating patients clinically diagnosed with AD dementia from those with other brain pathologies [17]. The latest CSF assay methods exhibit accuracy levels comparable to those outlined in Cochrane reviews.

Though tests founded upon the estimation of amyloid deposits reflecting amyloid metabolism deregulation demonstrate satisfactory sensitivity (81.5%), their specificity is compromised (66.5%) when identifying patients that will develop AD dementia symptoms. This phenomenon leads to non-AD patients being over-diagnosed with AD. Thus, labeling an AD patient solely based on deregulated amyloids or tau homeostasis is inappropriate, as not all individuals with such deregulation exhibit full AD symptoms [28,29,30,31,32,33]. Relying solely on biological amyloid markers for treatment prescriptions could consequently lead to excessive medication for non-AD patients. Ethically, this raises concerns about treating non-AD patients who will not respond to anti-amyloid treatments yet may be adversely affected by side effects.

To grasp the ramifications of amyloid peptide homeostasis deregulation in cognitively normal individuals, some studies have embarked on calculating the lifetime risk of AD dementia development in preclinical individuals. These individuals are defined as cognitively unimpaired, yet they present brain amyloidosis, as evidenced by a positive result in at least one amyloid assay [40]. The lifetime risks of AD dementia symptoms onset significantly vary based on age and gender. For example, a female with amyloidosis only faces lifetime risks of 8.4% at 90 years old and 29.3% at 65 years old. Thus, such preclinical individual have only a 30% lifetime risk of developing symptoms of AD dementia. It is worth noting that a woman without brain amyloidosis carries a lifetime risk of 18.7% at 65 years of age. This robustly establishes amyloid as a confirmed risk factor for AD dementia symptom development [40], which aligns with results from the recently published A4 Study clinical trial [41]. The inclusion criteria for patient selection in the A4 Study clinical trial necessitate participants meeting specific conditions. Their Mini-Mental State Examination (MMSE) score during screening must fall between 25 and 30. Their Global Clinical Dementia Rating (CDR) scale score during screening must be 0, indicating no cognitive impairment. Their Logical Memory II score during screening should range from 6 to 18. Additionally, the Florbetapir PET scan during screening must display evidence of brain amyloid pathology. Among participants, who had an average age of 72 years upon inclusion, only 32–35% demonstrated a cognitive decline of at least 0.5 CDR-SB points over the 240-week (4.5-year) clinical follow-up period. This implies that being amyloid positive in cognitively intact individuals is not a strong indicator of future cognitive AD decline. Moreover, according to the amyloid hypothesis, AD dementia onset is the inevitable outcome of Aβ accumulation if individuals live long enough to progress through the final stages of the cascade. However, the incidence of AD dementia in cognitively unimpaired individuals positive for both amyloid and tau CSF status (A+T+) aged 74 is below 20% after 5 years of follow-up and under 50% after 14 years [42]. This suggests that A+T+ status is not a robust proxy of cognitive AD decline. While it could be argued that the Aβ cascade is gradual and more extensive follow-up might approach a lifetime risk of 100%, current data do not confirm this hypothesis. Taken together, these findings confirm that amyloid and tau metabolism deregulation (identified through CSF analysis or PET imaging) constitutes a risk factor for AD dementia development [42] but falls short of confidently diagnosing individuals that will develop AD dementia symptoms (incomplete penetrance). 

Despite these findings, which suggest the necessity for more specific biomarker tests, research has predominantly concentrated on developing approaches based on core biomarkers that can nearly perfectly predict amyloid plaque presence in the brain over a patient’s lifetime. Accordingly, ongoing clinical studies have unveiled encouraging outcomes in the development of blood tests for non-invasive and universally applicable prediction of brain amyloid deposits. Plasma Aβ42/40 ratio assays have showcased remarkable areas under the receiver operating characteristic curve (AUROC) of up to 87%, thus accurately predicting the presence of amyloid plaques in the brain through various assays [35]. Consequently, blood levels of amyloid peptides can serve as a highly precise proxy for amyloid plaque presence in the brain of tested subjects. Similarly, plasma p-tau tests, especially p-tau 217 assays, have yielded AUROCs reaching up to 95% in distinguishing between MCI patients who are positive and negative for cerebral amyloidosis [43]. The ability of blood tests to predict amyloid metabolism deregulation is equally true for blood tests centered on the p-tau assay. For instance, a p-tau 217 blood test can predict, with a sensitivity of 93% and a specificity of 89%, amyloid-positive patients clinically diagnosed with AD dementia from amyloid-negative, clinically diagnosed patients with a dementia other than AD. However, this specificity drops to 47% when it comes to predicting amyloid-positive patients clinically diagnosed with AD dementia from amyloid-positive, clinically diagnosed patients with a dementia other than AD [44]. These data reaffirm that blood p-tau biomarkers, including plasma p-tau 217, serve more as predictors of amyloid status than as diagnostic tools for AD. Consequently, blood markers of amyloid or tau metabolism deregulation share the same specificity limitations as markers measured in the CSF or via PET imaging, thus corroborating the data presented in Cochrane’s reviews.

Nevertheless, despite the lack of specificity in the diagnostic performance of these markers for AD, a research framework proposing AD as a biological disease was introduced in 2018 [45]. These efforts have endorsed recommendations for diagnosing and characterizing AD strictly for research purposes. Central principles arising from these endeavors underscore the need to define AD biologically rather than relying on clinical symptoms. The condition is envisioned as a continuum commencing with the emergence of brain amyloidosis in preclinical individuals and advancing through stages of escalating pathological burden before culminating in the manifestation and progression of clinical symptoms. The diagnosis of AD is accomplished in vivo only through the identification of anomalies in core biomarkers. However, the practicality of this approach in clinical settings was subject to debate in 2021. This debate arises partly because some individuals exhibit these core biomarkers without developing AD symptoms. Moreover, these biomarkers may also be present in other brain diseases, which coexist with AD lesions as a comorbidity. The International Working Group recognizes these limitations in biomarker-based AD diagnosis and suggests their use be restricted to cases presenting specific AD phenotypes. Consequently, individuals who test positive for these biomarkers, yet do not exhibit cognitive impairment, should be classified as at risk rather than definitively diagnosed with AD [46].

In mid-2023, the Alzheimer’s Association decided to revise the NIA-AA framework 2018, and they unveiled their proposed revisions to clinical diagnosis guidelines at the Alzheimer’s Association International Conference for scientific input and review (https://aaic.alz.org/diagnostic-criteria.asp (accessed on 6 December 2023). This paradigm of defining neurodegenerative diseases based on their biological underpinnings rather than symptom presentations has evolved into a unifying concept applicable to all neurodegenerative conditions, thus extending beyond AD. The key update from the 2018 guidelines is to extend these diagnostic criteria not just within a research context, but also in routine diagnostic practice. The successive guidelines proposed for AD diagnosis seem to distill the condition down to the mere presence of biological markers, detached from the patient’s symptomatic presentation. As such, the deregulation of amyloid homeostasis is shifting from being a risk factor to constituting the definition of an AD patient within the medical community. 

In just over a century, the diagnostic approach to AD has undergone a significant evolution. Initially, it relied solely on clinical criteria, thus emphasizing a longitudinal analysis of a patient’s cognitive decline. Recently, however, there has been a shift towards exclusively biological criteria. These new criteria focus on biomarkers unrelated to the patient’s symptomatic presentation or its progression. Despite this shift, it is important to note that both experimental and clinical data have yet to fully endorse a diagnosis based solely on amyloid and tau biomarkers.

## 3. Interpretation of a Core Biomarker: Cholesterol against Amyloid Peptide (Figure 3)

In the field of medical research, significant parallels can be drawn between cholesterol in cardiovascular diseases and soluble amyloid peptide in AD, as their roles are similar in terms of risk factors and biomarkers. Serum cholesterol and low-density lipoprotein (LDL) are considered cardiovascular risk factors [47], while amyloid levels are increasingly regarded as definitive biomarkers of AD.

**Figure 3:**
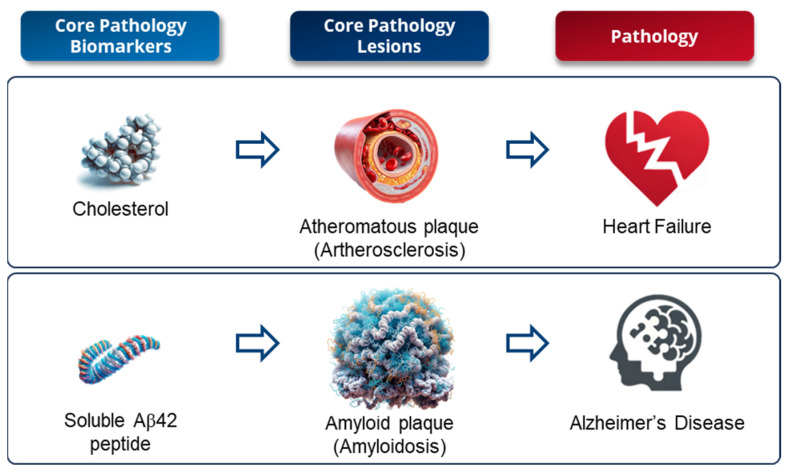
Comparison of core biomarkers used to identify people at risk of heart attack or Alzheimer’s disease. While there are obvious similarities between cholesterol as a core biomarker of a heart attack and the amyloid Aβ42 peptide as a core biomarker of AD, one is considered a risk factor of heart attacks while the other is becoming the ultimate marker for diagnosing AD.

Historical research has revealed that cholesterol is a key component of atherosclerotic plaques [48], thus enhancing our understanding of heart diseases. Similarly to cholesterol’s role in atherosclerosis, studies have shown that amyloid peptides are primary constituents of amyloid plaques in AD [2], thus enhancing our understanding of its pathology. However, while elevated levels of LDL-c or serum cholesterol are markers of atherosclerosis, serum cholesterol levels or the presence of atherosclerotic plaques are merely risk factors for the development of cardiovascular diseases [47]. Similarly, reduced levels of soluble amyloid in biofluids (CSF and blood) reliably indicate the presence of brain amyloid plaques, while levels of soluble amyloid in biofluids or amyloid plaques are risk factors for the onset of AD dementia symptoms [28,29,30,31,32,33]. Like cholesterol, the soluble peptide Aβ42 exerts physiological functions [49]. In both cases, it is a supra-physiological excess that is responsible for the pathological effects. Yet, it is important to note that the toxicity associated with an excess of soluble amyloid Aβ42 peptides is comparatively greater than that resulting from elevated blood cholesterol levels.

There will be significant differences in treatment strategies for heart diseases and AD. Statins, which are effective at lowering cholesterol levels, differ from anti-amyloid antibodies in terms of administration (oral vs. intravenous injection), cost (a few dozen dollars vs. over USD 20,000 annually), and side effects (few vs. significant side effects). Consequently, prescribing statins to as many hypercholesterolemia patients as possible is viewed as fulfilling a medical need and posing minimal risk to both the patient and the healthcare system. However, this widespread approach, which is feasible with statins, is not viable with anti-amyloid antibodies. Treating all amyloid-positive individuals is not feasible due to the high rate of over-medication (over 20%) associated with secondary risks and significant treatment costs. This underscores the need for precision medicine in AD focused on developing specific biomarkers for personalized and cost-effective therapeutic approaches. The aim is to manage AD more effectively and balance benefits and risks while considering the financial implications for healthcare systems.

## 4. A potential Confirmation Bias in the Search for Alzheimer’s Biomarkers

While existing studies have uniformly determined that amyloid-based diagnostic tests for AD lack specificity, the research community remains disproportionately focused on amyloid and tau species for AD diagnosis. This trend overlooks the potential long-term implications of this issue. It is hypothesized that such a persistent focus might be influenced by confirmation bias in the field.

Confirmation bias is an important concept in psychology and cognitive sciences. It refers to the human tendency to seek, interpret, and favor information that confirms pre-existing beliefs or hypotheses while ignoring, minimizing, or rejecting information that contradicts them. In other words, when faced with new information or experiences, the human mind tends to prefer those that align with our preconceived ideas and accepts them more easily. This can occur consciously or unconsciously, and it can influence decision making, the evaluation of evidence, and our perception of the world around us.

In this instance, once the diagnostic principles based on AD’s biological markers (indicative of amyloid metabolism homeostasis disruption and suggesting amyloid plaque presence) were established, researchers quickly adopted these criteria. They began using them for AD diagnosis in research settings, including patient selection for clinical trials, even before thoroughly assessing the diagnostic value of these criteria. Thus, in patients for whom AD symptoms are not suspected, analysis of core biomarkers linked to amyloid deposits is not considered a priority [50]. This makes it difficult for clinicians to estimate the specificity of core biomarkers in this cognitively impaired, non-AD population. For patients suspected of having AD because of their symptoms, biomarkers can be measured, but only as exclusion criteria. Amyloid-positive patients suspected of having AD will thus be diagnosed with AD, while amyloid-negative patients will be excluded for AD diagnosis. At the time of diagnosis, the clinician does not have the clinical elements—in particular, the cognitive decline towards more advanced stages that will follow the diagnosis—to establish the false-negative rate of amyloid biomarkers. Under these conditions, it remains complex for clinicians in their current practice to obtain an informed estimate of the use of biomarkers of deregulation of amyloid metabolism homeostasis. 

The addition of these biomarkers to the diagnostic decision-making process has increased confidence in the veracity of the diagnosis for physicians, as it is easier to interpret. Thus, the evaluation criterion became the diagnosis of truth: an AD patient must be amyloid-positive, and other brain pathologies must be amyloid-negative. This circular reasoning led to confirmation of the hypothesis that, based on these criteria, all patients diagnosed as non-AD were all negative and AD patients were all amyloid-positive in clinical routine. Although scientific data on the diagnostic performance of amyloid biomarkers were available, clinicians did not take them into account, as they were convinced of the veracity of the diagnosis based exclusively on the biomarkers to which they had access. Subsequently, the search for newer, more specific biomarkers appeared to take a secondary role, as biomarkers for amyloid deposits were deemed potentially adequate. However, this confirmation bias is far from trivial, and it could have important practical consequences for the search for new diagnostic biomarkers of pre-dementia AD.

A confirmation bias lies in the ability to analyze experimental data through the prism of pre-established hypothesis. The post-mortem analysis of brains of centenarians (aged between 100 and 111 years) without dementia symptoms revealed similar levels of amyloid and tau protein buildup as those seen in AD patients. Thus, 55% of the centenarians studied had an NIA amyloid stage score greater than or equal to 2 and 83% had a Braak NFT stage score greater than or equal to III. A large proportion of centenarians spontaneously present a deregulation of amyloid and tau metabolism homeostasis, resulting in the appearance of brain lesions characteristic of AD in the absence of any cognitive symptoms [51]. The presence of these lesions is thus not sufficient to make an AD diagnosis, which confirms the low specificity of these lesions to AD. In order not to invalidate the biological diagnosis, an argument put forward is that these people would have developed AD dementia if they had lived long enough. This argument rests on the principle that the lack of empirical evidence is not synonymous with empirical refutation. Therefore, the dependence on indemonstrable hypotheses tends to reinforce a pre-existing theory.

Demonstrating our hypothesis that confirmation bias may influence the perceived diagnostic utility of amyloid plaques in AD is difficult, yet it could provide some explanation for the prevailing tendency to attribute AD to its biological components despite known specificity constraints.

## 5. The Impact on the Search for AD Biomarkers and Treatments

### 5.1. Challenges in Precision Medicine: Treatment Complexity in Alzheimer’s Disease

The amyloid confirmation bias could have profound implications for the quest to identify diagnostic biomarkers for AD. The immediate consequence is a skewed focus on pinpointing biomarkers that align with amyloid status. As anti-amyloid treatments gain approval, AD management enters a precision medicine phase: administering the right drug to the right patient at the right time. Paradoxically, tests reliant on detecting amyloid deposits contradict the principles of precision medicine. They tend to mistakenly diagnose >20% of the patients without AD, thus leading to treatment for those who inherently will not respond to disease-modifying therapies (DMTs). While the impact of amyloid loads on memory issues in non-AD brain disorders is largely unexplored, scientific evidence challenges the notion that amyloid plaques are the primary cause of symptoms in these conditions. For instance, in disorders like Parkinson’s disease [52], Lewy body dementia [53], cortical basal syndrome [54], post-stroke neurodegeneration [55], schizophrenia [56], alcohol-related cognitive disorders [57], and late-life depression [58], which are all linked to mild cognitive impairment (MCI), the presence of amyloid deposits does not seem causally tied to memory problems. Instead, these deposits might stem from typical brain aging due to soluble amyloid clearance defects. Consequently, reducing amyloid levels in these patients might not effectively slow cognitive decline. The exclusive use of biological diagnosis to prescribe treatments, particularly anti-amyloid treatments, will insidiously tend to apply the same treatment to all amyloid-positive individuals, whether or not they have AD, and whether or not they respond to treatment.

### 5.2. Expanding Alzheimer’s Understanding beyond Amyloid: Challenges and Opportunities

Relying on a biological diagnosis rooted in amyloid plaque presence affects the efficacy demonstration of new treatments. Amyloid-based tests lead to the inclusion of non-responders in clinical trials due to low AD specificity, thus reducing statistical power in evaluating treatment effectiveness. To emulate cancer diagnosis successes, where highly specific early tests guide timely, tailored treatment [59], researchers must develop diagnostics that uphold this standard.

Reducing AD diagnosis to a mere biological definition constrains the discovery of unique AD pathological mechanisms. Studies driven by the biological definition compare amyloid-negative, cognitively normal subjects to amyloid-negative dementia patients, thus neglecting AD’s complexity. About 41% of cognitively normal, elderly patients test positive for amyloid spontaneously [60], and 15% of individuals clinically diagnosed with AD dementia are amyloid-negative [37]. Consequently, these studies elucidate amyloid-status-induced mechanisms rather than true AD mechanisms, thus reinforcing confirmation bias.

This confirmation bias oversimplifies AD complexity to amyloid and tau brain lesions. Amyloid metabolism deregulation is not binary but rather a nuanced spectrum. Even slight deregulation in an individual could trigger AD dementia without crossing arbitrary positivity thresholds, which underscores the need to explore resistance/resilience mechanisms against amyloid metabolism disruption. Understanding protective mechanisms could yield alternatives to anti-amyloid antibodies. Additionally, the focus on cerebral lesions disregards peripheral pathological mechanisms. Exploring peripheral mechanisms might unveil novel, lower-side-effect, anti-Alzheimer’s approaches. Indeed, the side effects associated with anti-amyloid antibodies, such as enlarged cerebral ventricles, cerebral edema, and microhemorrhages [61], may stem from their cerebral mode of action. These side effects could potentially arise from increased mechanical permeabilization of the blood–brain barrier due to heightened osmotic pressure [62] caused by a higher peripheral concentration of anti-amyloid antibodies relative to their cerebral concentration (<2% of the injected amount) [63]. Alternatively, they might result from an off-target effect on cerebral amyloid angiopathy (CAA), which could increase the Blood–Brain Barrier (BBB)’s permeability and cerebral edema [64]. Utilizing drugs with a primarily peripheral mode of action could mitigate these side effects, as their effectiveness does not depend on their presence in the brain, thereby preserving the integrity of the BBB. Furthermore, such drugs may allow for lower dosages compared to anti-amyloid antibodies, thus potentially reducing off-target biological effects. Developing peripherally acting drugs could potentially offer low-side-effect therapeutic options.

Finally, reducing AD diagnosis to a biological basis could hinder active treatment development at the asymptomatic stage. Using cognitive impairment absence and positive amyloid tests as inclusion criteria means only around 30% of participants in a primary prevention trial will suffer AD-related cognitive decline. With such a low responder rate, demonstrating cognitive decline reduction becomes highly unlikely.

## 6. Breaking out of Confirmation Bias

**Figure 4:**
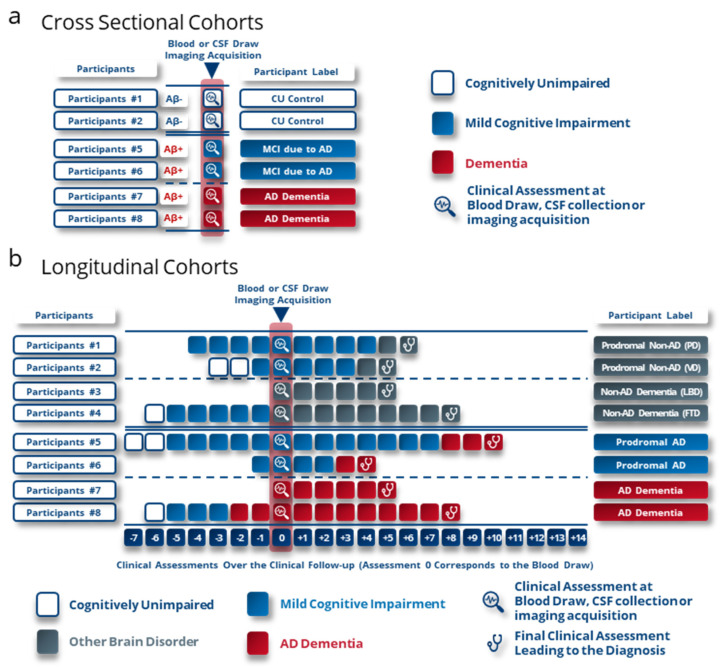
Labelling method proposed for participants in the discovery phases of new Alzheimer’s disease biomarkers to reduce the confirmation bias observed. (**a**) Current criteria for inclusion of participants in the discovery or validation phases of Alzheimer’s biomarkers. Confirmation bias is reflected here in these criteria, which guide the results and thus the biomarkers to be correlated with amyloid status and not with pathological status (AD or non-AD). (**b**) Proposed criteria for labelling participants in Alzheimer’s biomarker discovery or validation studies. These criteria are based on cohorts with longitudinal follow-up to enable labelling based on cognitive characterization of participants at an advanced stage of brain pathology. We also suggest not including cognitively unaltered participants as a reference group and instead including patients with brain pathologies other than AD to determine performance as a differential diagnosis. These were the criteria used in studies referenced in Cochrane reviews. PD, Parkinson’s Disease; VD, vascular dementia; LBD, Lewy body Dementia; FTD, frontotemporal dementia.

To counteract a potential confirmation bias and its detrimental impact on future research endeavors, we present a set of straightforward recommendations. At the outset, we emphasize the critical need to reevaluate participant inclusion criteria and categorization protocols in the initial phases of biomarker discovery phases. The practice of using cross-sectional clinical groups consisting of cognitively unimpaired (CU) individuals testing negative for amyloid, alongside individuals labeled as having dementia due to AD based on a cognitive impairment and amyloid positivity, poses a significant obstacle to unveiling novel biomarkers or panels specific for AD and not for amyloid status (Figure 4a). The hindrance arises from the fact that approximately 21% of participants are misdiagnosed with AD based solely on amyloid testing, thus impeding the identification of distinct biomarkers indicative of AD. 

To surmount this challenge, a viable solution involves categorizing patients according to their clinical diagnoses in the advanced stages of diverse brain disorders. Leveraging longitudinal cohorts becomes a pivotal strategy in this context (Figure 4b). One of the advantages of labeling patients based on a longitudinal cognitive decline lies in the alignment between the AD decline-related biomarkers and the need for clinical trials and the prescription of current or future treatments. Therefore, the discovery of new biomarkers based on retrospective cognitive labeling will highlight biomarkers that can identify patients who will progress towards AD dementia symptoms. This will improve the assessment of anti-Alzheimer’s therapies’ performance by including pre-demented patients who will progress towards AD dementia symptoms. These patients will also be the ones who should be prioritized for treatment compared to patients who will not experience cognitive decline (stable MCI) or who will develop symptoms other than AD dementia symptoms. Furthermore, the consideration of participant types is crucial. For robust identification of differential biomarkers of AD pre-dementia, it is essential to perform comparative analyses between CSF, blood or imaging biomarkers of individuals at the MCI stage, who have been followed longitudinally until they clinically progress to AD dementia or another neurodegenerative disorder (Figure 4b). This spectrum of cognitive disorders should encompass a wide range to encapsulate the utmost diversity within these alternative pathologies. In the discovery phase of new biomarkers, the array of disorders that could serve as a benchmark group includes, but is not limited to, frontotemporal dementia, Lewy body dementia, Parkinson’s disease, corticobasal degeneration, epilepsy, isolated amyloidosis, primary progressive aphasia, psychological or psychiatric disorders, traumatic brain injury, stroke, and vascular dementia. To ensure the discovery of broadly applicable biomarkers, it is advisable to undertake these investigative stages across a minimum of two independent cohorts, thereby mitigating the risk of identifying biomarkers with limited generalizability.

The identification of new biomarkers, made possible by unbiased labeling criteria, will facilitate a more accurate recognition of AD patients in the pre-dementia stages. These novel biomarkers or panel of biomarkers can be used in combination with amyloid status to prioritize patients for treatment with anti-amyloid antibodies for whom the risk of misdiagnosis is really low. They will also enable the development of new therapeutic strategies that are independent of an anti-amyloid mode of action. Additionally, they will lead to a more precise identification of AD patients even in the asymptomatic stage and could support the validation of primary prevention therapeutic approaches.

## 7. Alternative Biomarkers and More Possibilities

The development of novel imaging, CSF, or blood biomarkers that do not predict amyloid status but rather the cognitive progression towards symptoms of AD dementia would enable the implementation of the precision medicine needed to manage AD. Biomarkers not directly related to amyloid status are being evaluated, including, in particular, neuronal damage biomarkers (Neurofilament light chain protein (NfL), S100b and neuron-specific enolase (NSE)), biomarkers of neuro-inflammation (Glial fibrillary acidic protein (GFAP), Triggering receptor expressed on myeloid cells 2 (TREM2), chitinase 3-L1 (YKL-40), and Cytokines-chemokines) and other reactional biomarkers (Neurogranin) and markers of metabolic response (apolipoproteins, neurotrophic factors, intestinal and obesity markers, and diabetes and glycemic markers) [65]. However, most of these biomarkers are not specific to AD but are deregulated in the context of other neurodegenerative diseases [65]. This significantly reduces their potential as differential biomarkers.

An alternative is the discovery of multiomic biomarker panels (genomic, proteomic, lipidomic, and metabolic biomarkers). These biomarkers could then be used to train machine learning algorithms [66] capable of predicting which patients will develop symptoms of AD dementia from MCI or even an asymptomatic stage. The composition of these panels may vary and may potentially include or exclude amyloid peptides, p-tau proteins, or APOE genotyping. However, the development of such multiomic panels is still in nascent stages, with limited studies conducted to date.

One notable study identified a 10-metabolite panel (comprising PC diacyl aa C36:6, PC aa C38:0, PC aa C38:6, PC aa C40:1, PC aa C40:2, PC aa C40:6, PC acyl-alkyl ae C40:6, lysoPC a C18:2, Propionyl AC (C3), and C16:1-OH). Initially, plasma samples were taken from cognitively healthy individuals. These individuals were then clinically followed over several years to monitor cognitive changes. The study employed this metabolite panel to train an algorithm that successfully differentiated between those who remained cognitively unimpaired and those who converted to MCI or AD dementia with over 90% accuracy (AUC = 0.92, sensitivity/specificity of 90%/90%) [67]. However, this discovery was based on a single cohort without external validation. Subsequent validation in two independent cohorts, the Baltimore Longitudinal Study of Aging (BLSA) and the Age, Gene/Environment Susceptibility–Reykjavik Study (AGES-RS), did not replicate these findings. The analysis in these cohorts yielded significantly lower accuracy (BLSA, AUC = 0.642, sensitivity/specificity of 51.6%/65.7%; AGES-RS, AUC = 0.395, sensitivity/specificity of 47.0%/36.0%) [68]. Further machine learning analysis of 187 metabolite concentrations in the BLSA cohort indicated only moderate predictive value, which did not translate effectively to the AGES-RS samples [68]. These results underscore the importance of conducting the discovery phase of biomarker research using multiple independent cohorts to ensure the generalizability of findings and to minimize the risk of developing non-generalizable biomarker panels.

The potential of multiomic biomarker panels analyzed through artificial intelligence algorithms remains an underexplored avenue in AD research. This approach holds promise for identifying individuals with high specificity who are likely to develop cognitive symptoms of AD. Early identification would render these individuals eligible for targeted anti-AD treatments. Importantly, such a stratified approach could optimize the patient selection process by focusing on those who stand to benefit the most, thereby ensuring a favorable benefit-to-risk balance in therapeutic interventions. The clear advantage of this multiomic signature approach is that it could include biomarkers involved in various biological pathways, such as amyloid metabolism, tau metabolism, oxidative stress, inflammation, bioenergetics, blood coagulation, lipid metabolism, or immune response. This would not only enable the prediction of patients who are likely to develop AD dementia but also offer a tailored profile of each patient. This profile could identify the biological pathways specifically deregulated in each patient, thereby facilitating a more personalized therapeutic approach (likely a combination of treatments) best suited to their condition. However, a significant limitation in identifying such multiomic signatures is the challenge of accessing sufficient clinically followed patient samples until the onset of dementia during discovery phases. One potential solution to this obstacle of limited patient samples could be the pre-identification of biomarker panels in animal models, followed by their study in a smaller set of patient samples [69].

## 8. Conclusions

In conclusion, tracing the trajectory of Alzheimer’s diagnosis through history has illuminated a path of progress and challenges. From Dr. Alois Alzheimer’s discovery in 1906 to the current era of biomarker-driven diagnostics, our understanding of the disease has evolved significantly. The introduction of biomarkers, particularly those associated with amyloid metabolism deregulation, has offered valuable insights into the disease’s pathology. However, this journey has also been marked by the influence of a potential confirmation bias shaping research directions and potentially limiting the full comprehension of AD’s complexity. Confirmation bias, a psychological inclination to favor information that aligns with preconceived beliefs, has subtly steered the course of AD biomarker research. Despite scientific evidence indicating the limitations of amyloid-deposit-related markers due to specificity concerns, the focus has remained largely on these markers. This bias has hindered the exploration of more specific biomarkers and mechanisms, thus potentially overlooking significant nuances within the disease’s spectrum. Furthermore, the confirmation-bias-driven emphasis on amyloid markers risks over-diagnosis and over-medication of patients who may not truly have AD. As the landscape of precision medicine unfolds, it becomes crucial to embrace a more comprehensive approach that incorporates diverse biomarkers and clinical assessments to refine diagnoses and treatment strategies. Breaking free from confirmation bias necessitates a multi-faceted approach. Revisiting participant inclusion criteria and categorization protocols, employing retrospective cohorts, and embracing a wider range of cognitive disorders for comparison can offer a more holistic perspective on biomarker discovery. By broadening our view and accounting for the complexity of AD, we can navigate towards a more precise and comprehensive diagnostic framework. In the pursuit of diagnostic excellence, the amalgamation of diverse biomarkers, clinical evaluations, and an unwavering commitment to unbiased exploration are paramount. The future of AD diagnosis lies not in a single biomarker but in a symphony of insights that transcends the confines of confirmation bias. As we continue this journey, it is imperative that we draw on lessons from history, apply the best of scientific rigor, and navigate with an open mind to unlock the mysteries of AD for the benefit of patients, caregivers, and society at large.

## Figures and Tables

**Table 1:** Summary table of Cochrane reviews. This table encapsulates the conclusions drawn from each review, which uniformly indicate that the employment of these biomarkers as diagnostic tests is not recommended due to their inadequate specificity. Sensitivities above 80% are shown in bold.

**Cochrane** **reviews titles**	18F PET with florbetapir for the early diagnosis of Alzheimer’s disease dementia and other dementias in people with mild cognitive impairment (MCI)	18F PET with flutemetamol for the early diagnosis of Alzheimer’s disease dementia and other dementias in people with mild cognitive impairment (MCI)	18F PET with florbetaben for the early diagnosis of Alzheimer’s disease dementia and other dementias in people with mild cognitive impairment (MCI)	11C-PIB-PET for the early diagnosis of Alzheimer’s disease dementia and other dementias in people with mild cognitive impairment (MCI)	Plasma and cerebrospinal fluid ABeta42 for the differential diagnosis of Alzheimer’s disease dementia in participants diagnosed with any dementia subtype in a specialist care setting	CSF tau and the CSF tau/ABeta ratio for the diagnosis of Alzheimer’s disease dementia and other dementias in people with mild cognitive impairment (MCI)		Plasma and cerebrospinal fluid amyloid beta for the diagnosis of Alzheimer’s disease dementia and other dementias in people with mild cognitive impairment (MCI)		
**First author**	Martinez G.	Martinez G.	Martinez G.	Zhang S.	Kokkinou M.	Ritchie C.		Ritchie C.		
**Publication year**	2017	2017	2017	2014	2021	2017		2014		
**Reference**	[29]	[32]	[28]	[33]	[34]	[31]		[30]		
**Stage of AD progression**	MCI	MCI	MCI	MCI	Dementia	MCI		MCI		
**Type of Assay**	PET Imaging	PET Imaging	PET Imaging	PET Imaging	CSF Assay	CSF Assay	CSF Assay	CSF Assay	Plasma Assay	Plasma Assay
**Biomarkers**	florbetapir	flutemetamol	florbetaben	PIB	CSF Aβ42	p-tau	total tau	Aβ42	Aβ42/Aβ40	Aβ42
**Number of research** **papers**	3	1	1	9	13	15		14	1	1
**Numbers of Participants**	448	224	45	274	1704	1282		1349	562	565
**Sensitivity (%)**	67	64	**100**	**96**	79	**82**	77	**81**	**86**	**86**
**Specificty (%)**	71	69	**88**	58	60	47.5	72	64	70	50

## Data Availability

No new data were created.
